# Patterns in Benthic Biodiversity Link Lake Trophic Status to Structure and Potential Function of Three Large, Deep Lakes

**DOI:** 10.1371/journal.pone.0117024

**Published:** 2015-01-16

**Authors:** Barbara L. Hayford, Andrea M. Caires, Sudeep Chandra, Scott F. Girdner

**Affiliations:** 1 Department of Life Sciences, Wayne State College, Wayne, Nebraska, United States of America; 2 Biology Department, University of Nevada Reno, Reno, Nevada, United States of America; 3 Crater Lake National Park, Crater Lake, Oregon, United States of America; National University of Mongolia, MONGOLIA

## Abstract

Relative to their scarcity, large, deep lakes support a large proportion of the world’s freshwater species. This biodiversity is threatened by human development and is in need of conservation. Direct comparison of biodiversity is the basis of biological monitoring for conservation but is difficult to conduct between large, insular ecosystems. The objective of our study was to conduct such a comparison of benthic biodiversity between three of the world’s largest lakes: Lake Tahoe, USA; Lake Hövsgöl, Mongolia; and Crater Lake, USA. We examined biodiversity of common benthic organism, the non-biting midges (Chironomidae) and determined lake trophic status using chironomid-based lake typology, tested whether community structure was similar between the three lakes despite geographic distance; and tested whether chironomid diversity would show significant variation within and between lakes. Typology analysis indicated that Lake Hövsgöl was ultra-oligotrophic, Crater Lake was oligotrophic, and Lake Tahoe was borderline oligotrophic/mesotrophic. These results were similar to traditional pelagic measures of lake trophic status for Lake Hövsgöl and Crater Lake but differed for Lake Tahoe, which has been designated as ultra-oligotrophic by traditional pelagic measures such as transparency found in the literature. Analysis of similarity showed that Lake Tahoe and Lake Hövsgöl chironomid communities were more similar to each other than either was to Crater Lake communities. Diversity varied between the three lakes and spatially within each lake. This research shows that chironomid communities from these large lakes were sensitive to trophic conditions. Chironomid communities were similar between the deep environments of Lake Hövsgöl and Lake Tahoe, indicating that chironomid communities from these lakes may be useful in comparing trophic state changes in large lakes. Spatial variation in Lake Tahoe’s diversity is indicative of differential response of chironomid communities to nutrient enrichment which may be an indication of changes in trophic state within and across habitats.

## Introduction

Large, deep lakes are rare but provide an increasingly important source of water and food for human use and many serve as cultural icons (Crater Lake, USA; Lake Baikal, Russia). Lakes with depths greater than 100 m or surface areas greater than 248 km² comprise less than 0.0001% of the world’s lakes [1, 2], but support a significant amount of the world’s freshwater biodiversity, including 15% of the global diversity of freshwater fishes and 9% of non-insect freshwater invertebrates [3]. The benthic habitat of large lakes in particular supports at least 95% of all invertebrate species from large lakes and serves as an important component of whole-lake primary and secondary production [3].

Comparison studies between lakes have established linkages between anthropogenic impact and impairment of lake ecosystems, including changes in biodiversity [4]. Direct comparison of biodiversity is the basis of most biological monitoring for conservation research [5], but biodiversity comparisons between large, insular ecosystems such as lakes are difficult. Biogeographic patterns in species distribution may eclipse ecological patterns of distribution when comparing biodiversity of distant lake ecosystems [6, 7]. Using ubiquitous taxa in comparison studies may facilitate our understanding of changes to biodiversity of large lakes, particularly lakes facing impacts from human activities.

The objective of our study was to analyze the biodiversity of benthic communities from three large lakes: Lake Tahoe, USA; Lake Hövsgöl, Mongolia; and Crater Lake, USA. All three lakes are ecologically similar (e.g. deep and large, oligotrophic, alpine to subalpine, north temperate lakes) and represent different management strategies along a conservation gradient. We characterized benthic Chironomidae communities in these lakes. Chironomids are an excellent model organism for use in lake comparisons, particularly if study lakes are geographically distant from each other because they are widespread aquatic invertebrates [8] especially common in large lakes. For instance, Chironomidae from 14 of the world’s largest lakes represent close to 15% of global chironomid diversity [3]. Larval chironomids inhabit lake benthic environments from nearshore littoral zones to abyssal depths of the profundal zone [8] and are sensitive to eutrophication [9]. Their ubiquity and sensitivity allows them to be used in lake typology, an analytical method based on the presence or absence of species indicative of trophic states in aquatic ecosystems [10, 11, 12]. The specific objectives of this study were to: 1) determine the trophic status of the three lakes using chironomid-based lake typology; 2) test whether community structure was similar between the three lakes despite geographic distance; and 3) examine the variation of diversity between lakes and depth regions within each lake.

## Materials and Methods

### Study sites

Lake Tahoe is a subalpine, ultra-oligotrophic to oligotrophic, graben lake in the states of California and Nevada, western USA where increasing cultural eutrophication is coupling the flow of energy between the pelagic environment and benthic habitat where invertebrates reside (for a detailed description of the lake see [13, 14, 15]). Crater Lake is an alpine, ultra-oligotrophic, caldera lake in the state of Oregon, western USA (for a detailed description see [16]). Lake Hövsgöl is a subalpine, ultra-oligotrophic to oligotrophic, graben, lake in the Hövsgöl province in the north central region of Mongolia (for a detailed description of this location see [17, 18]). Morphometric characteristics for the three lakes are given in Table 1. Crater Lake is managed entirely by the United States Federal Government as part of the National Park System and is the most conserved of the three study lakes. It has experienced little to no cultural eutrophication. Lake Hövsgöl, located in south central Siberia, is managed by the Mongolian government as a National Park, which has allowed some natural resource use and development on the shores of the lake. It faces increasing eutrophication from grazing and tourism. Lake Tahoe has the greatest amount of development of the three lakes, and it has experienced gradual cultural eutrophication over the past four decades from extensive land use and urbanization in its watershed.

**Table 1:** Physical and geographic variables for the three study lakes.

	**Tahoe**	**Crater**	**Hövsgöl**
Max. length (km)	35	9.7	136
Max. width (km)	19	8	36.5
Max. depth (m)	501	595	262
Surface area (km²)	495	52.9	2760
Volume (km³)	151	18.7	381
Elev. (m asl)	1897	1883	1645
Watershed size (km²)	1320	60	4920
Average Secchi (m)	21	30.5	15.5
Latitude	39	42	51

### Sampling Methodology

**Figure 1:**
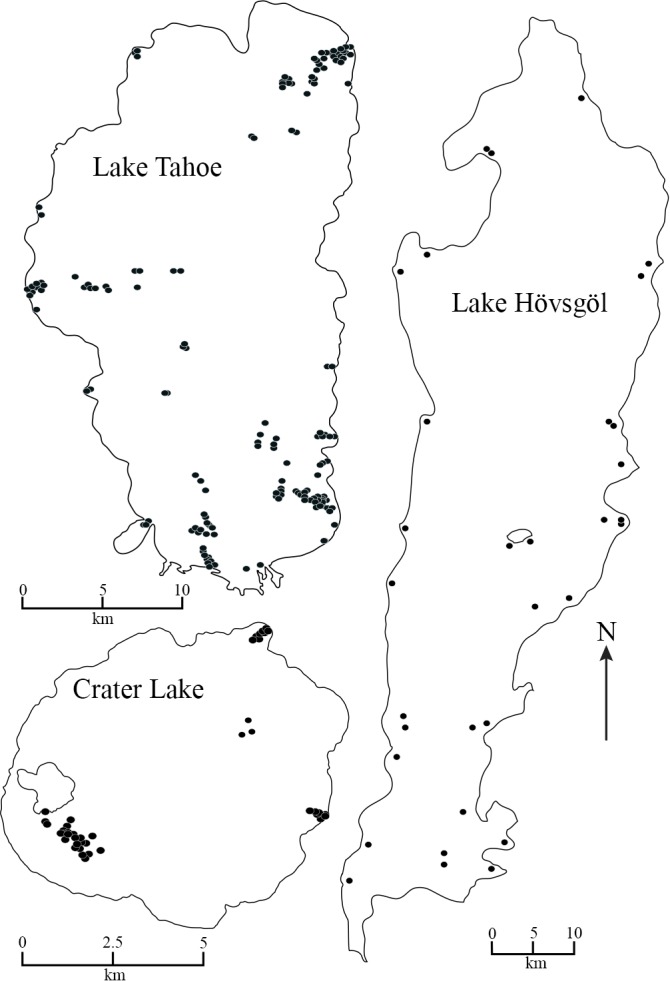
Map of Lake Tahoe, USA, Crater Lake, USA and Lake Hövsgöl, Mongolia. Solid circles indicate sampling locations in 2008 and 2009 for Lake Tahoe, 2009 for Crater Lake, and 1995–1997 for Lake Hövsgöl.

Benthic grab samples from Lake Tahoe were collected in 2008–09 from 4 transects that spanned nearshore to deep environments of the lake (1–450 m) and 6 short transects in the nearshore environment only (1–30 m; Fig. 1, S1 Table). Three to 5 replicate samples were collected at 5 m depth intervals from 1–100 m, 10 m depth intervals from 100–130 m, and 50 m depth intervals from 150–450 m. Collections occurred from June-September 2008 and March-July 2009 as weather permitted. Benthic grab samples were collected in Crater Lake in 2009 from 3 transects in soft substrate that spanned nearshore to deep environments (1–550 m; Fig. 1, S1 Table). Three to 5 replicate samples were collected at approximately 20 m depth intervals from 20–100 m, and 50 m depth intervals from 100–250 m in long transects. Several deep (450–550 m) collections were also made around the deepest area in the lake. Each sample from both Lake Tahoe and Crater Lake was collected using a Shipek grab (400 square cm) and washed through a 500-µm mesh bucket sieve. Substrates collected in the samples were visually assessed to determine the substrate composition as:% large rocks, particles larger than 256 mm; % cobble, particles from ~24 to 256 mm in diameter; % gravel, particles from ~ 2–64 mm in diameter; % sand, particles from ~ 0.06–2.0 mm in diameter; % silt, particles from ~ 0.004-.06 mm in diameter % clay, particles less than .004 mm in diameter with a slick and sticky feel; % macrophyte. Samples were elutriated (if necessary), and chironomids were handpicked for preservation. A sugar flotation and visual inspection method was employed to separate organisms from organic matter [19]. Chironomidae were preserved in 70% ethanol in the field, transferred to 95% ethanol in the lab where their head capsules were removed and slide mounted in Euparal slide mounting medium.

Benthic grab samples from Lake Hövsgöl were collected during 30 June to 20 July, 1995 from seven transects that spanned the nearshore to deep environment of the lake (1–260 m; Fig. 1, S1 Table) using a Ponar grab (900 square cm). Samples also were collected along two nearshore transects in the north end of the lake using a petite Ponar grab (225 square cm). Three to 8 replicate samples were collected from 5 m depth intervals from 1–100 m and 2–3 replicates at 10 m depth intervals from 100–260 m. Benthic grab samples from Lake Hövsgöl were collected also during 9–10 July of 1997 from 6 short transects in the nearshore environment (1–30 m) using a standard Ponar grab (900 square cm). Samples were washed through three sieves of decreasing sized mesh (top sieve mesh diameter = 2 mm; middle sieve mesh diameter = 500 µm; bottom sieve mesh diameter = 250 µm). Substrates collected in the samples were visually assessed to determine the substrate composition as: % large rocks, particles larger than 256 mm; % cobble, particles from ~24 to 256 mm in diameter; % gravel, particles from ~ 2–64 mm in diameter; % sand, particles from ~ 0.06–2.0 mm in diameter; % silt, particles from ~ 0.004-.06 mm in diameter % clay, particles less than. 004 mm in diameter with a slick and sticky feel; % macrophyte. Specimens were preserved in the field using 40% ethanol. Chironomidae were sorted from abiotic material and other macroinvertebrates and preserved in the lab with 95% ethanol and slide mounted in Euparal. Specimens from all three lakes were identified to genus, sub-genus or species group using keys by [20, 21].

Sampling permission was conditional upon funding by the California Tahoe Conservancy for Lake Tahoe and by the cooperative agreement between the Cooperative Ecosystems Studies Unit at the University of Nevada and Crater Lake National Park for Crater Lake and no further permission was necessary. Permission to sample Lake Hövsgöl was granted by Ministry of Nature and Environment through communication with Minister Z. Batjargal and D. Myagmarsuren, Head of the Department of Protected Areas. Sampling did not involve endangered or protected species.

### Data analysis

Only samples collected from soft substrates from the three lakes were used in analyses to reduce the confounding effect of substrate variation on densities and community composition. Chironomidae are common, abundant inhabitants of soft substrate [22]; thus, an analysis of soft substrates such as silt, mud, clay, and sand should capture most of the richness and community composition for the lakes. All abundance data were converted to density per square meter prior to analyses. Genera were used in comparison between the lakes to reduce the chance of variation arising from biogeographic effects on distribution of species. Ambiguous taxa were removed only if they were redundant with higher level taxa. For example, sometimes it is difficult to differentiate between *Cricotopus* and *Orthocladius* in the larval stage, in which case the specimens were assigned into the ambiguous group of *Cricotopus/Orthocladius*. If a site had specimens already assigned to either *Cricotopus* or *Orthocladius* and also had specimens assigned to *Cricotopus/Orthocladius* then the data for the ambiguous assignation of *Cricotopus/Orthocladius* were removed from analysis. If a site had specimens assigned to the *Cricotopus/Orthocladius* group, but not to either of the genera separately, then the data in the ambiguous grouping were retained for analysis. This process allows for retention of high quality diversity data without biasing analyses with redundant data [23].

Comparisons between the three lakes were broken down into chironomid communities from nearshore and deep environments as these two types of habitats have very different benthic communities [22, 24, 25, 26]. Nearshore environment for these analyses is defined as habitat from 3.5m-25m. This habitat range in depth is similar to the nearshore environment defined for Lake Tahoe as 1–30 m [26] and as 1to approximately 20 m [27]. Samples from 26–29 m were removed from analyses to reduce the chance of communities overlapping between habitat categories, increasing the likelihood of formation of discrete categories. Samples collected at depths less than 3.5 m were not included in the analyses because Crater Lake and Lake Hövsgöl did not have a comparable number of samples in these depths. The deep environment for these analyses is defined as 30–65 m, with 65 m being the maximum depth at which chironomids were collected with 3 or more species present for community analyses in all three lakes. The deep environment represents the sublittoral to upper profundal zone in Lake Hövsgöl [25], the sublittoral zone in Crater Lake, and the sublittoral zone in Lake Tahoe [27].

Different numbers of samples were collected from each lake and lake depth zone, leading to a concern that differences in sampling effort would affect community analysis. However, many more samples were collected than were included in this study, particularly for Crater Lake. Examination of these samples indicated that Crater Lake has low chironomid diversity (data not shown). We performed rarefaction analysis on the chironomid communities for each lake zone. The total number of taxa reached the asymptote for the randomization routine prior to reaching the total number of samples collected at each depth zone for this study, indicating that sampling effort was adequate in estimating diversity. Thus we deemed that no rarefaction adjustment was required for this study.

Lake typology analysis was performed to determine the trophic status of the three lakes following established methods [10, 28] for the Chironomidae assemblages collected from the deep zone. This method of lake typing has been used for decades as an initial step in describing lake conditions. Chironomid genera and species were run through the typology key [10] to type each lake. Note that the key can be used with a mixture of genus and species level taxa [10]. Ordination of study sites based on Chironomidae was used to test whether community structure was similar between the three lakes and lake depths (e.g. nearshore and deep environments) despite geographic distance. Mean densities were log (x+1) transformed and were then analyzed using non-metric multi-dimensional scaling (nMDS) analysis. Stress values were used to evaluate goodness of fit [29]. The results were visualized in a scatter plot. To test for significant differences in chironomid communities, a one-way analysis of similarity (ANOSIM) was calculated based on Bray-Curtis dissimilarities, using nearshore and deep environments for each lake as grouping categories. ANOSIM is a multivariate procedure that compares distances within groups to distances between groups such that communities may be considered different if their within-group distances are significantly smaller than their between-group distances [30]. ANOSIM produces an R statistic. The closer R is to 1, the greater the distance or dissimilarity between two communities. The resulting distance matrix was run through a randomization routine with 9999 permutations to calculate a significance value.

The Shannon-Wiener diversity index was calculated for nearshore and deep chironomid communities in each lake to examine the variation of diversity between depth zones and lakes. We tested for significant differences in chironomid diversity between nearshore and deep environments from the three lakes using a one-way analysis of variance (ANOVA). We used a general linear model ANOVA due to unequal sample sizes in nearshore and deep environments, followed by Tukey’s test to determine pairwise differences in diversity.

Shannon-Wiener diversity, and ANOSIM were formed using PAST (version 2.16). One-way general linear model ANOVAs were run on Number Crunching Statistical Software (version 3.18 Kaysville Utah). Significance for all analyses was set at *p* ≤ 0.05. Rarefaction analysis was run using EstimateS (version 9.1.0).

## Results

Lake typology based on Chironomidae indicator taxa from Lake Tahoe, Crater Lake, and Lake Hövsgöl samples indicate three different trophic classes for the lakes. Lake Hövsgöl was classified as α-oligotrophic (ultra-oligotrophic) based on the presence of *Pseudodiamesa*. Crater Lake was classified as δ-oligotrophic (oligotrophic) based on dominance of *Heterotrissocladius subpilosus* group. Lake Tahoe was classified as ζ-Oligotrophic to η-mesotrophic (oligotrophic/mesotrophic), depending on whether specific species of *Tanytarsus, Monodiamesa,* and *Paracladopelma* are present.

**Figure 2:**
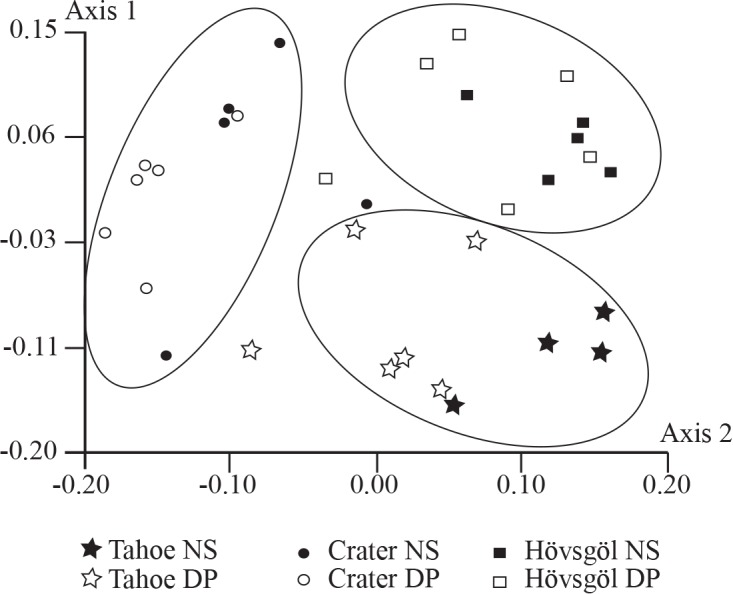
Ordination based on non-metric multidimensional scaling (nMDS) analysis of chironomid communities from the three lakes and lake zones (black fill and NS = nearshore, no fill and DP = deep zone) in this study. Two axes were chosen to best display the data (stress = 0.20).

Study sites clearly separated by lake and depth zone along the first and second axes in the nMDS ordination (Fig. 2). Crater Lake sites separated along the first axis. Lake Hövsgöl and Lake Tahoe sites separated along both axes. Lakes and depth zones are relatively discrete with some overlap within each lake, particularly for Crater Lake (Fig. 2). Less sites overlap between lakes, though some sites may be observed close together between lakes (Fig. 2). The ANOSIM supports results of the ordination analysis. In total, communities were significantly distant from each other with an overall R of 0.45 (*p* < 0.001) and pairwise comparisons indicated that about half of the communities were significantly different (Table 2). Sites within lakes were not significantly different nor were sites from nearshore zones in Lake Tahoe when compared to either Crater Lake and Lake Hövsgöl or the deep zone of Lake Hövsgöl (Table 2). The Lake Tahoe deep zone was not significantly different than the community in the Crater Lake deep zone and the Crater Lake nearshore communities were not significantly different when compared to either community in Lake Hövsgöl (Table 2).

**Table 2:** Pairwise comparisons of chironomid communities from the different habitats in Lake Tahoe, Crater Lake, and Lake Hövsgöl.

**Group pairwise comparison**		**ANOSIM**	
Lake and habitat	Lake and Habitat	*R*	Significance
Lake Tahoe nearshore	Lake Tahoe deep	0.28	*p <* 0.01
Lake Tahoe nearshore	Crater Lake nearshore	0.50	*p <* 0.01
Lake Tahoe nearshore	Crater Lake deep	0.56	*p <* 0.01
Lake Tahoe nearshore	Lake Hövsgöl nearshore	0.30	*p <* 0.01
Lake Tahoe nearshore	Lake Hövsgöl deep	0.03	NS
Lake Tahoe deep	Crater Lake nearshore	0.17	*p <* 0.01
Lake Tahoe deep	Crater Lake deep	0.29	*p <* 0.01
Lake Tahoe deep	Lake Hövsgöl nearshore	0.47	*p <* 0.01
Lake Tahoe deep	Lake Hövsgöl deep	0.14	NS
Crater Lake nearshore	Crater Lake deep	0.32	*p <* 0.01
Crater Lake nearshore	Lake Hövsgöl nearshore	0.59	*p <* 0.01
Crater Lake nearshore	Lake Hövsgöl deep	0.89	*p <* 0.01
Crater Lake deep	Lake Hövsgöl nearshore	0.65	*p <* 0.01
Crater Lake deep	Lake Hövsgöl deep	0.92	*p <* 0.01
Lake Hövsgöl nearshore	Lake Hövsgöl deep	0.03	NS

Distance R-statistics and *p-*values given from ANOSIM. The closer R is to 1 the greater the distance or dissimilarity between two communities being compared.

A total of 51 taxa of Chironomidae were identified from 5 subfamilies for this study (S2 Table). Crater Lake was characterized by a depauperate fauna and lacked two of the subfamilies that were collected from the other two lakes (S2 Table). Richness varied between the three lakes and lake environments. Lake Tahoe had the greatest richness with 35 genera, followed by Lake Hövsgöl with 27 genera, and Crater Lake with 12 genera. Nearshore environments supported a greater number of taxa than did deep environments in each lake (S2 Table).

**Figure 3:**
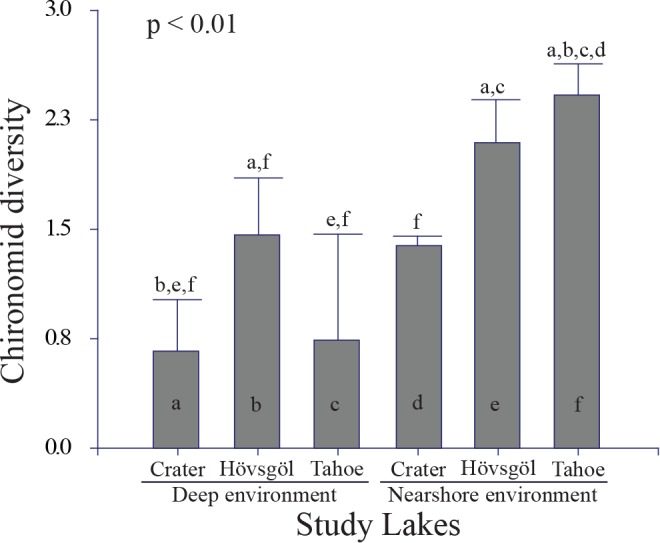
Variation in mean (± 1 SE) taxon diversity of chironomid communities in this study. The y axis represents Shannon-Wiener diversity (H’). The x axis represents lake zones and lakes. Letters that differ above and within the bars indicate a significant difference in mean diversity (H’) (*P* < 0.05; one-way ANOVA, Tukey’s test) for chironomid communities collected from the different lake regions and lakes.

Mean Shannon-Wiener diversity of chironomid communities was 0.93 in Crater Lake, 1.43 in Lake Hövsgöl, and 1.67 in Lake Tahoe and varied significantly between Lake Hövsgöl and Crater Lake (One-way ANOVA*p* <0.05, F = 5.05). Mean diversity varied between the depth zones of the three lakes, with the highest diversity in Lake Tahoe’s nearshore community and the lowest in the Crater Lake deep environment (one-way ANOVA, *p* < 0.00, F = 12.30, Fig. 3). Chironomid diversity in the Lake Tahoe nearshore was significantly higher than diversity in all other lake zones with the exception of the Lake Hövsgöl nearshore community, and Crater Lake’s deep environment had significantly lower diversity than the communities in the Lake Hövsgöl deep and nearshore environments (Fig. 3).

## Discussion

Results of the lake typology analysis corroborate pelagic indicators classifying Lake Hövsgöl as ultra-oligotrophic [18]. The results indicating that Crater Lake is oligotrophic are similar, though not identical to the classification of Crater Lake as ultra-oligotrophic based on chemical and optical studies [31]. On the other hand, the lake typing of Lake Tahoe as oligotrophic/mesotrophic differs considerably from the traditional pelagic classification of the lake as ultra-oligotrophic to oligotrophic [13, 14]. The outcome of chironomid-based lake typology analysis of Lake Tahoe is supported by other studies showing impact to lake benthos by gradual eutrophication. Nutrient enrichment in Lake Tahoe over the past 40 years has led to changes in energy flow between pelagic and benthic communities [15] and significant declines in overall benthic taxa including major changes in chironomid communities [26]. The differences we observed between benthic and pelagic measures of lake trophic condition based on benthic invertebrate community change may indicate a substantial shift in trophic state, while snapshot pelagic and nutrient indicators indicate only a gradual change. Thus, understanding changes to benthic environments may be a critical aspect of documenting functional changes in the lake that may not be otherwise signaled from pelagic measurements and could be the result of the increased coupling or decoupling of habitats.

The structure of chironomid communities exhibited a surprisingly low degree of dissimilarity, given the large geographic distance between Lake Hövsgöl and the other two lakes and the different structure, origin, and age of Crater Lake [32]. These results indicate that Crater Lake and Lake Hövsgöl may potentially serve as a reference for changes in Lake Tahoe, particularly changes to Lake Tahoe’s deep zones (e.g. [26]). The similar age, origin, and specifically the similar morphometry shared by Lake Hövsgöl and Lake Tahoe [13, 14, 17, 18], and the relative proximity of Crater Lake and Lake Tahoe may explain the low degree of dissimilarity between chironomid communities. Caution should be used in interpreting these results since ANOSIM was used here only to determine the relative degree of dissimilarity and these are three very large and complex lakes. We do suggest that the utility of Lake Hövsgöl and Crater Lake as a reference lake for Lake Tahoe should be further explored.

Diversity varied between depth zones within and between the study lakes. We observed the lowest nearshore chironomid diversity in Crater Lake, followed by Lake Hövsgöl, then by Lake Tahoe. This indicates that nearshore chironomid diversity increased along a gradient of increasing shoreline development. The diversity of benthic invertebrates has been shown to increase with increased nutrients in mesocosm studies [33], streams [34], and lakes [4, 35], which could explain the differences in diversity among our study lakes. Greater habitat heterogeneity and surface area are factors that also support greater diversity [3, 4]. This may help explain the lower chironomid diversity observed in Crater Lake. However, Lake Tahoe has less surface area and a smaller watershed than does Lake Hövsgöl, and we would thus expect lower diversity in Lake Tahoe than Lake Hövsgöl according to lake surface area and heterogeneity. It is therefore more likely that the differences in chironomid diversity observed between Lakes Hövsgöl and Tahoe are a result of increased nutrient inputs along Lake Tahoe’s shoreline. Diversity of macroinvertebrates increases with nutrient loading, but typically declines past a nutrient threshold [34, 36]. The higher diversity of Lake Tahoe’s chironomid community in the nearshore zone may actually betoken impairment, particularly in oligotrophic systems that naturally support relatively low diversity [4].

Nearshore and deep zones exhibited markedly different patterns in diversity in our study lakes. Contrary to the patterns observed in the nearshore environment, diversity in the deep zones was highest in Lake Hövsgöl followed by Lake Tahoe, which had comparably low diversity relative to Crater Lake (Fig. 3). The low chironomid diversity in Lake Tahoe’s deep environment, relative to the deep environment of Lake Hövsgöl in particular, corroborates observed density declines and changes to community structure in the Lake Tahoe basin over time [26]. Significant declines in densities of Lake Tahoe’s deep-environment benthic invertebrates over the past four decades may be attributed to changes to the lake environment, including gradual eutrophication, declines in the spatial extent of native macrophytes, and the impact of non-native species [26].

## Conclusions

Our research indicates that nearshore chironomid communities were different from deep-zone chironomid communities within and between the three lakes. Variation in community structure between the depth zones within each lake implies that spatial variation by depth should be a consideration in biodiversity-based research, particularly in biological assessment and monitoring (e.g. [37, 38]). The research also shows that Chironomidae may serve in the comparison of ecologically-similar large, deep lakes across large geographic distances. This study is a first step toward establishing methods for monitoring benthic communities from the world’s large, deep lakes. As reference lakes are identified we may eventually be able to create metrics such as the benthic quality index developed for lakes in Europe [39, 40]. Currently, Lake Tahoe faces stress from dense shoreline development. Increased development is beginning to encroach in Lake Hövsgöl as well, while Crater Lake remains relatively free of environmental stressors. Comparing biodiversity at such large scales may assist in lake management of these unique lake systems. Collectively, three important outcomes of our research for conservation and management of lakes were clear: 1) these large lake benthic invertebrate communities are sensitive to trophic conditions and benthic monitoring should accompany pelagic measures of lake trophic state, 2) large lakes may serve as benthic community references for one another, even across large geographic distances, and 3) depth variation in benthic communities can be substantial and must be considered in monitoring programs and diversity comparisons. Given that benthic communities are often overlooked in lake management, we suggest that lake managers use early indicators of eutrophication, such as changes in benthic diversity and lake-typology to monitor large lakes.

## Supporting Information

S1 Table Geographic coordinates for the field study sites. Some site location coordinates for Crater Lake were lost after making the map in Fig. 1. Other missing data are not mapped on Fig. 1.
(DOCX)

S2 Table Community structure and species richness of Chironomidae by lake and lake zone (NS = nearshore zone, DP = deep zone). (DOCX)

S1 File Supporting table includes chironomid density per square meter for each sample and site location coordinates for samples. (XLSX)
