# Supplementary material for: Patterns in Benthic Biodiversity Link Lake Trophic Status to Structure and Potential Function of Three Large, Deep Lakes
Source: PLoS One. 2015 Jan 16;10(1):e0117024. doi: 10.1371/journal.pone.0117024 (PMC4296932; doi:10.1371/journal.pone.0117024)
Supplement: S1 Table — Some site location coordinates for Crater Lake were lost after making the map in Fig. 1. Other missing data are not mapped on Fig. 1. (DOCX) [file pone.0117024.s001.docx]

Electronic Table S1. Geographic coordinates for the field study sites. Note that geographic coordinates were lost for some locations used to create the maps in Figure 1.

| Site Code | Lake | Depth zone | Latitude | Longitude |
| --- | --- | --- | --- | --- |
| CB26 | Tahoe | TahoeDP | 39.21589 | -119.936122 |
| CB29 | Tahoe | TahoeDP | No data | |
| CB30 | Tahoe | TahoeDP | 39.215256 | -119.935887 |
| CB34 | Tahoe | TahoeDP | 39.215271 | -119.936004 |
| CB39 | Tahoe | TahoeDP | 39.214707 | -119.935876 |
| CB48 | Tahoe | TahoeDP | 39.214256 | -119.93586 |
| MARLA19 | Tahoe | TahoeDP | 38.992287 | -119.959857 |
| MARLA20 | Tahoe | TahoeDP | 38.992261 | -119.95981 |
| MB16 | Tahoe | TahoeDP | 39.09204 | -120.159543 |
| MB17 | Tahoe | TahoeDP | 39.091849 | -120.15947 |
| MB29 | Tahoe | TahoeDP | 39.093604 | -120.159758 |
| MB30 | Tahoe | TahoeDP | No data | |
| MB44 | Tahoe | TahoeDP | 39.093456 | -120.16033 |
| MB46 | Tahoe | TahoeDP | No data | |
| MB57 | Tahoe | TahoeDP | No data | |
| SL21 | Tahoe | TahoeDP | 38.970507 | -119.962192 |
| SL24 | Tahoe | TahoeDP | 38.970059 | -119.961724 |
| SL34 | Tahoe | TahoeDP | 38.971969 | -119.962807 |
| SL35 | Tahoe | TahoeDP | 38.97143 | -119.962369 |
| SL36 | Tahoe | TahoeDP | 38.971818 | -119.962351 |
| SL37 | Tahoe | TahoeDP | 38.971669 | -119.962564 |
| ZC19 | Tahoe | TahoeDP | 39.008538 | -119.959149 |
| Carnelian1 | Tahoe | TahoeNS | 39.221481 | -120.0806 |
| Carnelian2 | Tahoe | TahoeNS | No data | |
| Carnelian3 | Tahoe | TahoeNS | No data | |
| Carnelian4 | Tahoe | TahoeNS | 39.223586 | -120.081196 |
| Carnelian5 | Tahoe | TahoeNS | No data | |
| Carnelian6 | Tahoe | TahoeNS | No data | |
| CB1 | Tahoe | TahoeNS | 39.217604 | -119.931017 |
| CB10 | Tahoe | TahoeNS | 39.221042 | -119.929689 |
| CB11 | Tahoe | TahoeNS | 39.221042 | -119.929712 |
| CB2 | Tahoe | TahoeNS | 39.217627 | -119.931157 |
| CB3 | Tahoe | TahoeNS | 39.217398 | -119.930939 |
| CB4 | Tahoe | TahoeNS | 39.217234 | -119.930654 |
| CB5 | Tahoe | TahoeNS | 39.218692 | -119.930379 |
| CB7 | Tahoe | TahoeNS | 39.220114 | -119.930438 |
| CB8 | Tahoe | TahoeNS | 39.220079 | -119.929313 |
| CB9 | Tahoe | TahoeNS | 39.221048 | -119.929817 |
| CR1 | Tahoe | TahoeNS | 39.045502 | -119.949532 |
| CR2 | Tahoe | TahoeNS | No data | |
| CR3 | Tahoe | TahoeNS | 39.045771 | -119.949578 |
| CR4 | Tahoe | TahoeNS | 39.045454 | -119.952164 |
| CR5 | Tahoe | TahoeNS | No data | |
| CR6 | Tahoe | TahoeNS | No data | |
| CR7 | Tahoe | TahoeNS | 39.045532 | -119.952976 |
| CR8 | Tahoe | TahoeNS | No data | |
| ELK4 | Tahoe | TahoeNS | 38.98669 | -119.962071 |
| Emerald1 | Tahoe | TahoeNS | 38.963704 | -120.083152 |
| Emerald2 | Tahoe | TahoeNS | No data | |
| Emerald3 | Tahoe | TahoeNS | No data | |
| Emerald4 | Tahoe | TahoeNS | 38.96229 | -120.084249 |
| Emerald5 | Tahoe | TahoeNS | No data | |
| Emerald6 | Tahoe | TahoeNS | No data | |
| Emerald7 | Tahoe | TahoeNS | 38.962454 | -120.084669 |
| Emerald8 | Tahoe | TahoeNS | No data | |
| Emerald9 | Tahoe | TahoeNS | No data | |
| MARLA1 | Tahoe | TahoeNS | 38.992968 | -119.956099 |
| MARLA12 | Tahoe | TahoeNS | 38.992079 | -119.959167 |
| MARLA15 | Tahoe | TahoeNS | 38.992622 | -119.959074 |
| MARLA2 | Tahoe | TahoeNS | 38.99305 | -119.955687 |
| MARLA4 | Tahoe | TahoeNS | 38.992918 | -119.956997 |
| MARLA5 | Tahoe | TahoeNS | 38.992982 | -119.958016 |
| MARLA6 | Tahoe | TahoeNS | 38.992697 | -119.958581 |
| MARLA7 | Tahoe | TahoeNS | 38.992718 | -119.958847 |
| MARLA8 | Tahoe | TahoeNS | 38.992578 | -119.959026 |
| MARLA9 | Tahoe | TahoeNS | 38.992225 | -119.959081 |
| MB1 | Tahoe | TahoeNS | 39.092877 | -120.160619 |
| MB11 | Tahoe | TahoeNS | 39.091475 | -120.160363 |
| MB2 | Tahoe | TahoeNS | 39.091721 | -120.160469 |
| MB3 | Tahoe | TahoeNS | 39.091848 | -120.160522 |
| MB6 | Tahoe | TahoeNS | 39.091563 | -120.160267 |
| MB9 | Tahoe | TahoeNS | No data | |
| Meek1 | Tahoe | TahoeNS | 39.037531 | -120.120432 |
| Meek3 | Tahoe | TahoeNS | No data | |
| Meek4 | Tahoe | TahoeNS | 39.037422 | -120.120401 |
| Meek5 | Tahoe | TahoeNS | No data | |
| Meek6 | Tahoe | TahoeNS | No data | |
| Meek7 | Tahoe | TahoeNS | 39.037455 | -120.12062 |
| Meek8 | Tahoe | TahoeNS | No data | |
| Meek9 | Tahoe | TahoeNS | No data | |
| RICH 10 | Tahoe | TahoeNS | 38.94248 | -120.040625 |
| RICH 11 | Tahoe | TahoeNS | 38.942848 | -120.040921 |
| RICH 13 | Tahoe | TahoeNS | No data | |
| RICH 14 | Tahoe | TahoeNS | 38.945189 | -120.042323 |
| RICH 15 | Tahoe | TahoeNS | 38.945246 | -120.042783 |
| RICH 8 | Tahoe | TahoeNS | No data | |
| RICH 9 | Tahoe | TahoeNS | No data | |
| SH1 | Tahoe | TahoeNS | 39.201065 | -119.931672 |
| SH2 | Tahoe | TahoeNS | No data | |
| SH3 | Tahoe | TahoeNS | 39.201092 | -119.931685 |
| SH4 | Tahoe | TahoeNS | 39.045502 | -119.949532 |
| SL1 | Tahoe | TahoeNS | 38.967396 | -119.954504 |
| SL10 | Tahoe | TahoeNS | 38.969281 | -119.959037 |
| SL11 | Tahoe | TahoeNS | 38.969304 | -119.958461 |
| SL12 | Tahoe | TahoeNS | 38.968763 | -119.957792 |
| SL2 | Tahoe | TahoeNS | 38.966826 | -119.954249 |
| SL3 | Tahoe | TahoeNS | 38.966947 | -119.953389 |
| SL5 | Tahoe | TahoeNS | 38.968325 | -119.955523 |
| SL6 | Tahoe | TahoeNS | 38.968577 | -119.955892 |
| SL7 | Tahoe | TahoeNS | 38.968321 | -119.957081 |
| SL8 | Tahoe | TahoeNS | 38.968811 | -119.958013 |
| SL9 | Tahoe | TahoeNS | 38.969474 | -119.95923 |
| TB10 | Tahoe | TahoeNS | No data | |
| TB12 | Tahoe | TahoeNS | 39.134855 | -120.151252 |
| TB3 | Tahoe | TahoeNS | 39.177715 | -120.123977 |
| TB7 | Tahoe | TahoeNS | 39.163163 | -120.135922 |
| ZC10 | Tahoe | TahoeNS | 39.008452 | -119.954019 |
| ZC11 | Tahoe | TahoeNS | 39.008423 | -119.954837 |
| ZC3 | Tahoe | TahoeNS | 39.007855 | -119.950553 |
| ZC4 | Tahoe | TahoeNS | 39.007975 | -119.951158 |
| ZC5 | Tahoe | TahoeNS | 39.008008 | -119.951656 |
| ZC6 | Tahoe | TahoeNS | 39.008151 | -119.952401 |
| ZC7 | Tahoe | TahoeNS | 39.008087 | -119.952456 |
| ZC8 | Tahoe | TahoeNS | 39.008109 | -119.952273 |
| ZC9 | Tahoe | TahoeNS | 39.008432 | -119.953048 |
| Zephyr 9 | Tahoe | TahoeNS | No data | |
| Zephyr1 | Tahoe | TahoeNS | No data | |
| Zephyr2 | Tahoe | TahoeNS | No data | |
| Zephyr3 | Tahoe | TahoeNS | No data | |
| Zephyr4 | Tahoe | TahoeNS | 39.00757 | -119.954017 |
| Zephyr5 | Tahoe | TahoeNS | No data | |
| Zephyr6 | Tahoe | TahoeNS | 39.007662 | -119.953905 |
| 401 | Hövsgöl | HövsgölDP | 50.5833333 | 100.3586111 |
| 403 | Hövsgöl | HövsgölDP | 50.5691667 | 100.3586111 |
| 408 | Hövsgöl | HövsgölDP | 50.7405556 | 100.2819444 |
| 412 | Hövsgöl | HövsgölDP | 50.7072222 | 100.4425000 |
| 416 | Hövsgöl | HövsgölDP | 50.8888889 | 100.5391667 |
| 431 | Hövsgöl | HövsgölDP | 51.3100000 | 100.7602778 |
| 432 | Hövsgöl | HövsgölDP | 51.2969444 | 100.7466667 |
| 440 | Hövsgöl | HövsgölDP | 51.1208333 | 100.3261111 |
| 443 | Hövsgöl | HövsgölDP | 51.1177778 | 100.6852778 |
| 449 | Hövsgöl | HövsgölDP | 50.9947222 | 100.6733333 |
| 455 | Hövsgöl | HövsgölDP | 51.5161111 | 100.6238889 |
| 456 | Hövsgöl | HövsgölDP | 51.5161111 | 100.6244444 |
| 461 | Hövsgöl | HövsgölDP | 51.4480556 | 100.4522222 |
| 402 | Hövsgöl | HövsgölNS | 50.6350000 | 100.3952778 |
| 404 | Hövsgöl | HövsgölNS | 50.7508333 | 100.2788889 |
| 406 | Hövsgöl | HövsgölNS | No data | |
| 418 | Hövsgöl | HövsgölNS | 50.9447222 | 100.2522222 |
| 435 | Hövsgöl | HövsgölNS | 51.3027778 | 100.2663889 |
| 438 | Hövsgöl | HövsgölNS | 51.1175000 | 100.3233333 |
| 444 | Hövsgöl | HövsgölNS | 51.1144444 | 100.6925000 |
| 445 | Hövsgöl | HövsgölNS | 51.0658333 | 100.7072222 |
| 448 | Hövsgöl | HövsgölNS | 50.9952778 | 100.7108333 |
| 452 | Hövsgöl | HövsgölNS | No data | |
| 454 | Hövsgöl | HövsgölNS | No data | |
| 458 | Hövsgöl | HövsgölNS | No data | |
| 460 | Hövsgöl | HövsgölNS | 51.4544444 | 100.4425000 |
| 469 | Hövsgöl | HövsgölNS | near 51.5923 | near 100.5749 |
| 470 | Hövsgöl | HövsgölNS | near 51.5923 | near 100.5749 |
| 471 | Hövsgöl | HövsgölNS | near 51.5923 | near 100.5749 |
| 482 | Hövsgöl | HövsgölNS | No data | |
| 483 | Hövsgöl | HövsgölNS | 50.9680556 | 100.5211111 |
| 486 | Hövsgöl | HövsgölNS | 50.9641667 | 100.4877778 |
| 488 | Hövsgöl | HövsgölNS | 50.7355556 | 100.4097222 |
| 489 | Hövsgöl | HövsgölNS | 50.7030556 | 100.2591667 |
| 490 | Hövsgöl | HövsgölNS | 50.5955556 | 100.2041667 |
| 491 | Hövsgöl | HövsgölNS | 50.5486111 | 100.1680556 |
| 493 | Hövsgöl | HövsgölNS | 50.4966667 | 100.1947222 |
| 97003 | Hövsgöl | HövsgölNS | 50.9163889 | 100.2569444 |
| 97008 | Hövsgöl | HövsgölNS | 50.9163889 | 100.2569444 |
| 97010 | Hövsgöl | HövsgölNS | 50.9163889 | 100.2569444 |
| 97011 | Hövsgöl | HövsgölNS | 50.9847222 | 100.2800000 |
| 97014 | Hövsgöl | HövsgölNS | 50.9847222 | 100.2800000 |
| 97018 | Hövsgöl | HövsgölNS | 50.9847222 | 100.2800000 |
| 97024 | Hövsgöl | HövsgölNS | 50.9913889 | 100.7080556 |
| 97026 | Hövsgöl | HövsgölNS | 50.9913889 | 100.7080556 |
| 97029 | Hövsgöl | HövsgölNS | 50.9913889 | 100.7080556 |
| 97034 | Hövsgöl | HövsgölNS | 50.8975000 | 100.6036111 |
| 97040 | Hövsgöl | HövsgölNS | 50.8975000 | 100.6036111 |
| 97042 | Hövsgöl | HövsgölNS | 50.5625000 | 100.4502778 |
| 97045 | Hövsgöl | HövsgölNS | 50.5625000 | 100.4502778 |
| 97047 | Hövsgöl | HövsgölNS | 50.5625000 | 100.4502778 |
| 97053 | Hövsgöl | HövsgölNS | 50.8975000 | 100.6036111 |
| 97054 | Hövsgöl | HövsgölNS | 50.5961111 | 100.4830556 |
| 97057 | Hövsgöl | HövsgölNS | 50.5961111 | 100.4830556 |
| 97060 | Hövsgöl | HövsgölNS | 50.5961111 | 100.4830556 |
| CLB28 | Crater | CraterDP | 42.931592 | -122.141116 |
| Cleet 10 | Crater | CraterDP | No data | |
| Cleet 12 | Crater | CraterDP | No data | |
| Cleet 6 | Crater | CraterDP | No data | |
| Cleet 7 | Crater | CraterDP | No data | |
| Cleet 8 | Crater | CraterDP | No data | |
| Cleet 9 | Crater | CraterDP | No data | |
| Spring 10 | Crater | CraterDP | No data | |
| Spring 4 | Crater | CraterDP | No data | |
| Spring 5 | Crater | CraterDP | No data | |
| Spring 6 | Crater | CraterDP | No data | |
| Spring 7 | Crater | CraterDP | No data | |
| Spring 8 | Crater | CraterDP | No data | |
| Spring 9 | Crater | CraterDP | No data | |
| CLB2 | Crater | CraterNS | 42.978193 | -122.077528 |
| CLB4 | Crater | CraterNS | 42.978257 | -122.077588 |
| CLB6 | Crater | CraterNS | No data | |
| CLB7 | Crater | CraterNS | No data | |
| CLB8 | Crater | CraterNS | No data | |
| Cleet 4 | Crater | CraterNS | 42.97841 | -122.077623 |
| Spring 3 | Crater | CraterNS | No data | |
| Spring1 | Crater | CraterNS | No data | |
