# Supplementary material for: Patterns in Benthic Biodiversity Link Lake Trophic Status to Structure and Potential Function of Three Large, Deep Lakes
Source: PLoS One. 2015 Jan 16;10(1):e0117024. doi: 10.1371/journal.pone.0117024 (PMC4296932; doi:10.1371/journal.pone.0117024)
Supplement: S2 Table — (DOCX) [file pone.0117024.s002.docx]

Electronic Table S2. Community structure and species richness of Chironomidae by lake and lake zone (NS=nearshore zone, DP=deep zone).

| Taxon | TahoeNS | TahoeDP | CraterNS | CraterDP | HövsgölNS | HövsgölDP |
| --- | --- | --- | --- | --- | --- | --- |
| TANYPODINAE |  |  |  |  |  |  |
| *Ablabesmyia* | x |  |  |  |  |  |
| *Conchapelopea* |  |  |  |  | x |  |
| *Paramerina* |  |  | x |  |  |  |
| *Procladius* | x | x |  | x | x | x |
| *Thienemannimyia* | x |  |  |  | x | x |
| DIAMESINAE |  |  |  |  |  |  |
| *Potthastia longimana* group |  |  |  |  | x | x |
| *Protanypus* |  |  |  |  | x | x |
| *Pseudodiamesa* |  |  |  |  | x | x |
| PRODIAMESINAE |  |  |  |  |  |  |
| *Monodiamesa* | x | x |  |  | x | x |
| ORTHOCLADIINAE |  |  |  |  |  |  |
| *Corynoneura* |  |  |  |  | x |  |
| *Cricotopus* | x |  | x | x | x |  |
| *Cricotopus/Orthocladius/Paratrichocladius* |  | x |  |  |  | x |
| *Heterotrissocladius* *subpilosus* group | x |  | x | x |  |  |
| *Heterotrissocladius* *marcidus* group | x |  |  |  | x | x |
| *Orthocladius* |  |  | x | x | x |  |
| *Paracladius* | x |  |  |  | x | x |
| *Parakiefferiella* | x |  |  |  |  |  |
| *Psectrocladius (Mesopsectrocladius)* | x |  |  |  |  |  |
| *Psectrocladius (Psectrocladius)* | x |  | x | x | x | x |
| *Pseudosmittia* |  |  |  |  | x |  |
| *Rheocricotopus* |  |  |  |  | x |  |
| *Stilocladius* |  |  |  |  |  | x |
| *Synorthocladius* |  |  | x |  |  |  |
| *Thienemanniella* | x |  |  |  |  |  |
| *Zalutschia* |  |  |  |  | x |  |
| CHIRONOMINAE: CHIRONOMINI |  |  |  |  |  |  |
| *Chironomus* | x | x |  | x | x |  |
| *Cladopelma* | x |  |  |  |  |  |
| *Cryptochironomus* | x | x |  |  |  |  |
| *Cryptotendipes* | x |  |  |  |  |  |
| *Dicrotendipes* | x |  |  |  |  |  |
| *Endochironomus* | x | x |  | x |  |  |
| *Lauterborniella* | x |  |  |  |  |  |
| *Microchironomus* | x |  |  |  |  |  |
| *Microtendipes* |  |  |  |  | x |  |
| *Paracladopelma doris* group | x | x | x |  | x | x |
| *Paralauterborniella* | x |  |  |  |  |  |
| *Paratendipes albimanus* | x |  |  |  |  |  |
| *Phaenopsectra* | x | x | x | x |  |  |
| *Polypedilum (Polypedilum)* | x | x | x |  | x | x |
| *Robackia* | x |  |  |  |  |  |
| *Saetheria* | x |  |  |  |  |  |
| *Sergentia* |  |  |  |  | x |  |
| *Stictochironomus* | x |  |  |  | x | x |
| CHIRONOMINAE: PSEUDOCHIRONOMINI |  |  |  |  |  |  |
| *Pseudochironomus* | x |  |  |  |  |  |
| CHIRONOMINAE: TANYTARSINI |  |  |  |  |  |  |
| *Cladotanytarsus* | x | x |  |  |  |  |
| *Constempellina* |  |  |  |  | x |  |
| *Micropsectra* | x | x |  |  | x | x |
| *Paratanytarsus* |  |  |  |  | x | x |
| *Rheotanytarsus* | x |  |  |  |  |  |
| *Stempelinella* | x |  |  |  |  |  |
| *Tanytarsus spp.* | x | x |  |  | x | x |
| Richness by habitat zone | 34 | 12 | 8 | 8 | 26 | 17 |
| Total richness by lake | 35 | | 12 | | 27 | |
